# Ovariectomy and High Fat-Sugar-Salt Diet Induced Alzheimer's Disease/Vascular Dementia Features in Mice

**DOI:** 10.14336/AD.2024.03110

**Published:** 2024-10-01

**Authors:** Sahar Sweetat, Moti Ben Shabat, Paschalis Theotokis, Nir Suissa, Eleni Karafoulidou, Olga Touloumi, Rami Abu-Fanne, Oded Abramsky, Gilly Wolf, Ann Saada, Amit Lotan, Nikolaos Grigoriadis, Hanna Rosenmann

**Affiliations:** ^1^Department of Neurology, The Agnes Ginges Center for Human Neurogenetics, Hadassah Hebrew University Medical Center, Jerusalem, Israel; ^2^Faculty of Medicine, The Hebrew University of Jerusalem, Jerusalem, Israel. Hadassah BrainLabs-National Knowledge Center for Research on Brain Diseases, Hadassah-Hebrew University Medical Center, Jerusalem Israel.; ^3^Department of Neurology, AHEPA University Hospital, Aristotle University of Thessaloniki, Greece.; ^4^Department of Clinical Biochemistry, Hadassah Hebrew University Medical Center, Jerusalem, Israel.; ^5^Biological Psychiatry Laboratory, Hadassah Hebrew University Medical Center, Jerusalem Israel Faculty of Medicine, The Hebrew University of Jerusalem, Jerusalem, Israel.; ^6^Department of Psychology, School of Psychology and Social Sciences, Achva Academic College, Be’er Tuvia, Israel.; ^7^Department of Genetics, Hadassah Hebrew University Medical Center, Jerusalem, Israel; Faculty of Medicine, The Hebrew University of Jerusalem, Jerusalem, Israel.

**Keywords:** Alzheimer's disease, sporadic mouse model, high fat-sugar-salt diet, ovariectomy, dementia, vascular dementia

## Abstract

While the vast majority of Alzheimer's disease (AD) is non-familial, the animal models of AD that are commonly used for studying disease pathogenesis and development of therapy are mostly of a familial form. We aimed to generate a model reminiscent of the etiologies related to the common late-onset Alzheimer's disease (LOAD) sporadic disease that will recapitulate AD/dementia features. Naïve female mice underwent ovariectomy (OVX) to accelerate aging/menopause and were fed a high fat-sugar-salt diet to expose them to factors associated with increased risk of development of dementia/AD. The OVX mice fed a high fat-sugar-salt diet responded by dysregulation of glucose/insulin, lipid, and liver function homeostasis and increased body weight with slightly increased blood pressure. These mice developed AD-brain pathology (amyloid and tangle pathologies), gliosis (increased burden of astrocytes and activated microglia), impaied blood vessel density and neoangiogenesis, with cognitive impairment. Thus, OVX mice fed on a high fat-sugar-salt diet imitate a non-familial sporadic/environmental form of AD/dementia with vascular damage. This model is reminiscent of the etiologies related to the LOAD sporadic disease that represents a high portion of AD patients, with an added value of presenting concomitantly AD and vascular pathology, which is a common condition in dementia. Our model can, thereby, provide a valuable tool for studying disease pathogenesis and for the development of therapeutic approaches.

## INTRODUCTION

Alzheimer's disease (AD) is the most common neurodegenerative dementia disease, characterized by cognitive impairments with the presence of: depositions of amyloid-β (Aβ), the toxic fragment of the degradation of the amyloid precursor protein (APP), refered as amyloid plaques; intracellular aggregation of the tau microtubule associated protein, particularly in its hyperphosphorylated state, refered as neurofibrillary tangles; mitochondrial dysfunction; neuroinflammation (microglia and astrocytes are recruited and activated by the presence of amyloid plaques and tau pathology, as a neuroprotection mechanism, yet their activation causes a chronic neuroinflammatory process that lead to neurodegeneration) and neuronal loss within the brain [1]. While the familial form of the disease, the early onset form [due to mutations in the amyloid precursor protein gene (APP), presenilin 1 gene (PSEN1) and presenilin 2 gene (PSEN2)], is very rare, the sporadic presentation is of late-onset (LOAD), including 95% of the cases [2]. Sporadic disease is a complex disorder associated with various risk factors. Aging is the main one; others include the female sex [3] and various acquired risk factors, including diabetes, hypertension, obesity, and dyslipidemia [4, 5]. Genetic susceptibility is also implicated in the development of sporadic AD [2]: low frequency variants with intermediate effects, such as the cholesterol carrier apolipoprotein E isoform (APOE), implicated in numerous AD-related processes including crosstalk with Aβ, tau phosphorylation, mitochondrial dysfunction and neuroinflammation [6], or variants of the triggering receptor expressed on myeloid cells 2 (TREM2), implicated in microglial clearance of aggregation-prone proteins [7]; and common variants conferring a small increased risk for AD, such as the phosphatidylinositol-binding clathrin assembly protein (PICALM), implicated in clearance of amyloid and tau pathology, with the genetic variants affecting its expression [8], or clusterin (CLU), an extracellular chaperone, acting as a Aβ chaperone and interacts with several AD- related proteins [9].

Although sharing similarities in brain pathology and cognitive impairment, as well as in CSF biomarkers - the familial form (due to mutations in the amyloid-related genes) and the LOAD are not identical entities. They differ in features such as age at onset (<50 years in familial, ≥65 in LOAD), sex distribution (equal in familial form, higher in females in LOAD), and also differ in amyloid and tau burden, and rate of brain atrophy [10].

While the vast majority of cases are non-familial AD, the animal models of AD that are in common use for studying disease pathogenesis and development of therapy are mainly of a familial form [11]. Although the efficacy of various drugs for AD has been shown in those animals, they were not effective in the clnic [12, 13]. A model more reminiscent of the etiologies related to the LOAD sporadic disease, the common form of AD, for studying disease pathogenesis and for the development of therapeutic approaches, is currently lacking.

We aimed to generate a non-familial AD mouse model with a broad spectrum of characteristics of the sporadic form. As AD pathology commonly coexists with that of the vascular dementia in the elderly, and it seems that most patients are actually affected by mixed dementia (AD and vascular) [14, 15], we were also interested in following the development of vascular damage in the brain of the AD mouse model. Our rationale was to expose naïve mice concomitantly to various risk factors related to LOAD. We included the main contributors, aging and female sex, together with the acquired (environmental) AD risk factors that are also associated with vascular diseases: diabetes, hypertension, obesity, and dyslipidemia [4, 5, 16]. To address the risk factors of aging and female sex, female mice underwent ovariectomy (OVX) to accelerate aging by inducing menopause, which depletes the neuroprotective ovarian hormones [17]. Ovariectomy allowed us to generate an aged (postmenopausal) status without requiring the mice to reach old age. C57Bl mice were fed a high fat-sugar-salt diet (components of Western Diet) to address the acquired/environmental AD risk factors. Our results show that ovariectomized mice fed a high the fat-sugar-salt diet showed increased serum glucose, insulin, and lipids, with impaired cognitive performance, accompanied by brain pathology of amyloid and tangles, with gliosis and vascular impairment. The development of AD features in mice under AD-related environmental risk factors may provide a tool to be used as a model for the LOAD sporadic disease, the common form of AD/dementia, having an advantage over modeling the rare mutations genetic form.

## MATERIALS AND METHODS

### Mice

C57Bl/6J-Rcc female mice were exposed from the age of 2 months for about 6.5 months to a high fat-sugar-salt diet [TelKad Custom Diet TD.150158 containing by weight approximately: fat (21%; 0.2% cholesterol), carbohydrates (44%, with 30% sucrose), protein (17.3%) and salt (4%). Components as gr/Kg included: Casein 195.0, sucrose 304.05, corn starch 150.0, Anhydrous Milklfat 210.0, Cholesterol 1.5, cellulose 50.0, mineral mix 35.0, calcium carbonate 4.0, vitamin mix 10.0, antioxidant 0.04 and sodium chloride 37.41] and 1% NaCl in drinking water. After 2 months, mice went through an OVX operation (these mice termed: " OVX-Diet mice"). Control mice were fed with standard mouse diet [TelKad 2918, containing: fat (6%, from soybean oil), carbohydrates (44%, from wheat and corn), protein (18%)], and went through sham operation, which included the abdominal incision, and closure of the incision. Mice were housed in equally sized groups in 42.5 × 26.2 × 18.5 cm cages in an SPF-certified facility. Food and water were provided ad libitum. Room temperature was 22±2°C. All measurements were performed during the light phase of a 12 h light-dark cycle (lights on at 07:00). The wellbeing of the mice was routinely monitored.

All experiments were approved by the Institutional-Ethics-Committee of The Hebrew-University of Jerusalem.

**Figure 1.**
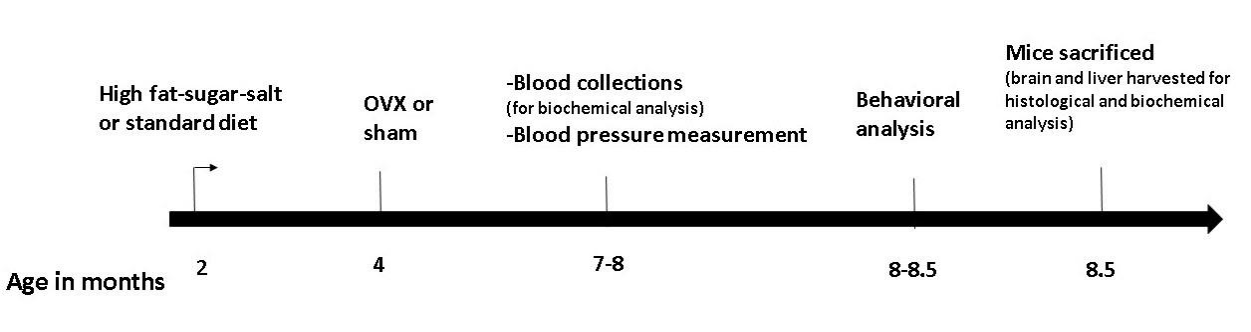
Study Design.

Animals were weighed once a week. Blood was collected from the facial vein at time points during the 5-6 months following starting of the diet, and the serum was used for biochemical analysis. Blood pressure levels were taken at 5.5 months following starting of the diet, and behavioral analysis were performed at 6-6.5 months following starting of the diet, and then the mice were sacrificed. The brain and liver were harvested for biochemical or histological studies (Study Design in Fig. 1).

### Noninvasive blood pressure measurement

Blood pressure levels were taken using the clinically validated Volume Pressure Recording (VPR) sensor technology (CODA® Kent scientific, RRID: SCR_018585) on a group of 4-5 mice per experimental group. The tail of the mouse was used for the measurements, and an animal nose cone holder was used to ensure accurate reading while the mice were awake. Measurements from 10 consecutive readings (using the 5-10 informative ones) were averaged for each mouse. Briefly, mice were placed in restraint tubes and left for 20 min to warm (tail skin surface temperature ~ 30 °C) on the instrument warming platform, and a pressure transducer was placed on the mouse’s tail. To prevent stress effects, mice were allowed to habituate to this procedure and train before measurements were performed.

### Biochemical analysis of the serum

Glucose level in serum was tested using the Glucose Assay kit (Abcam, ab65333). Insulin was measured using the Ultra-Sensitive Mouse Insulin ELISA (Crystal Chem, 90080). Levels of liver enzymes Aspartate amino-transferase (AST), and Alanine aminotransferase (ALT) were tested using SimpleStep ELISA® Kits (Abcam, ab263882 Mouse AST and ab282882 Mouse ALT, respectively). Total cholesterol and low-density lipoproteins (LDL) were measured (on pooled serum samples) using the the Atellica CH Analyzer, Siemens Heathineers.

### Behavioral studies

All measurements were performed during the light phase of a 12h light-dark cycle, during about 2-6 hours after lights switched on. Six months following starting of the diet (which was about 4 months following OVX), the mice went through cognitive tests:

#### Y maze

This test evaluates short-term memory. The Y-maze is a three-arm maze with all arms at equal angles, 30 cm in length and 5 cm in width with walls 12 cm high. Mice were initially placed in the middle, and the sequence of arm entries were recorded and analyzed with EthoVision for each mouse over an 8-min period. The triads with all three arms represented (i.e., ABC, CAB, or BCA but not ABB) - were considered 'correct triads' [18, 19]. Correct triad ratio was calculated as number of correct triads divided by total triads made. Visiting the same arm twice in a single triad (i.e. ABA or CAC) was considered as a mistake (termed "Revisit"), while visiting the same arm twice in a row (i.e., AAB or CCB) was considered as a greater mistake (termed "Direct revisit"). Direct revisits were calculated as number divided by total arm visits.

#### Novel Object Recognition (NOR)

The novel object recognition test is used to evaluate cognition, especially non-spatial recognition memory, and is considered a hippocampal dependent test [20- 23]. On the training day, the animals were placed in a 25 × 25 cm arena containing two identical objects for 10 minutes and then returned to their home cages. On the testing day, 24 hours later, the mice placed in the same open arena, with one familiar object and one novel object, different in shape, color, and texture from the familiar one. Each mouse was allowed to explore the arena for 4 minutes. The ratio of exploration of the novel object and the total exploration of the two objects, as duration and frequency, were calculated and presented in the figure. The test was performed using the Ethovision 10 system, providing fully computerized, blinded and unbiased measurement. Normal animals tent to explore the novel object longer time and frequency than the familiar one, indicating normally long-term recognition memory [19, 24-26].

#### Open field

This test evaluates animal motor activity as well as anxiety levels. The apparatus consisted of a square arena measuring 50 × 50 × 33 cm under 15 lux illuminations. Mice were allowed to explore the arena for 6 min, while their location was tracked and recorded by a video camera positioned overhead. Using Ethovision (Noldus Information Technologies, Wageningen, The Netherlands), distance moved by the animal, its velocity, time spent in the central zone of the arena (10 × 10 cm) was extracted [27].

### Mitochondrial enzymatic assays

Enzymatic assays were performed in homogenates of cortex and liver samples. The activity of the Cytochrome c oxidase (COX, complex 4 of the electron transfer chain) activity was measured by monitoring the oxidation of reduced cytochrome c at 550 nm [28]. Citrate synthase (CS) - ubiquitous mitochondrial matrix Krebs-cycle enzyme (used as a mitochondrial marker enzyme), was measured in the presence of acetyl-CoA and oxaloacetate by monitoring the liberation of CoASH coupled to to 5′,5′-dithiobis (2-nitrobenzoic) acid at 412 nm. Measurements were performed in a double beam spectrophotometer UVKON XS (Secomam France). Enzymatic activities (nmol/min/mg protein) were expressed as COX, CS and ratio (normalized to CS activity), presented as value relative to controls (ratio).

### Histology and immunohistochemistry (IHC)

Mice were anesthetized with Ketamine/Domitor and perfused via the ascending aorta with ice-cold PBS. Brains and livers were harvested. One half of the brain was fixed in 4% paraformaldehyde for 16-20 h at 4 °C for histological studies, and the other half was stored at -80°C for biochemical analysis. Brain hemispheres (hemi-section) were further appropriately processed, dehydrated in graded ethanol baths (50-95%) and cleared in xylene solution before being embedded in paraffin for sagittal sectioning at 7 μm. Immunohistochemistry was performed on adjacent serial sections of paraffin according to standard procedures [29]. Briefly, brain sections were deparaffinized in xylene, hydrated in alcohol baths and incubated in oxygen peroxide solution in order to block endogenous peroxidase enzyme. Then, sections were incubated in antigen retrieval solution. Blocking step in 10% FBS (Fetal Bovine Serum) was required to prevent non-specific binding. Afterwards, followed overnight incubation with primary antibodies for quantification of amyloid pathology (anti- -amyloid; 28365, Santa Cruz), tangle pathology (anti- AT8 and anti- AT180; MN1020, MN1040, Thermo) [30-32]. astroglial cells (anti- GFAP; 20334, DAKO), and microglia (anti- Iba-1; 019-19741, Waco), amyloid pathology (anti-β-amyloid; 28365, Santa Cruz), tangle pathology (anti- AT8 and anti- AT180; MN1020, MN1040, Thermo). Microglial activation state was quantified by a morphometric analysis determining the ramification index (RI), which represents a ratio of the projection area (cell perimeter) to the cell soma. Activation of microglia induces morphological changes from a highly ramified cell shape with long dendriform branches and many ramifications (high RI) to an activated phenotype with amoeboid cell shape (low RI) [33]. Analysis of microglial microphotographs of Iba-1 positive microglia was acquired with ImageJ software (Skeletonize and Fractal analysis plugin tools).

Sections were silver-impregnated by the Gallyas-silver method that stains tangles and nerve cell processes fine fibrils containing the abnormal tau protein in AD and tauopathies [30, 34, 35]. For staining of fibrillar amyloid we used thioflavin-S, a dye which binds to the characteristic β-pleated sheet conformation of amyloid [36, 37]. Paraffin-embedded brain sections were used for thioflavin-S staining. In brief, tissue sections were deparaffinized in xylene and then rehydrated in a series of graded alcohol baths. Sections were then incubated in filtered 1% aqueous Thioflavin-S (T1892, Sigma) solution protected from light at room temperature. After incubation, slides were washed with distilled water, dehydrated in alcohol baths and coversliped in aqueous mounting media. Slides were stored in dark conditions in 4°C until microscopic evaluation. Microscopic analysis and image acquisition was performed on a fluorescent microscope (Zeiss Axioplan II).

Blood vessels were stained using the biotinylated lectin-Ab (anti- lectin; L-0651, Sigma) [38] with specific immunostaining protocol as reported previously [31]. The next day, sections were incubated with secondary antibodies such as goat anti-rabbit IgG (BA1000, Vector) and goat anti-mouse (BA9200, Vector). For vascular alteration we also used the rabbit anti-VEGF (ABS82, Sigma-Aldrich) and the secondary was anti-rabbit IgG (Vector). Then, sections were incubated with avidin and DAB (3,3'-diaminobenzidine) solutions. Hematoxylin was used as counterstain for nuclei.

In every IHC stain procedure each Ab is validated, in order to distinguish genuine target staining from background. Briefly, to verify antibody specificity, one section was always used as a negative control for each experimental group. In these terms, negative control sections receive the exact same blocking and secondary Ab buffer as all other slides, but they did not receive primary antibodies.

For the basic neuropathological study, all sections were analyzed under 40× optical fields. Sections were observed with a light microscope (Zeiss) and images were captured with a camera (Nikon) for histology evaluation. The total number of cells was counted, and data were expressed as cells per square millimeter. All histological measurements were performed using the Image J software analysis (Fiji., NIH, USA).

**Figure 2.**
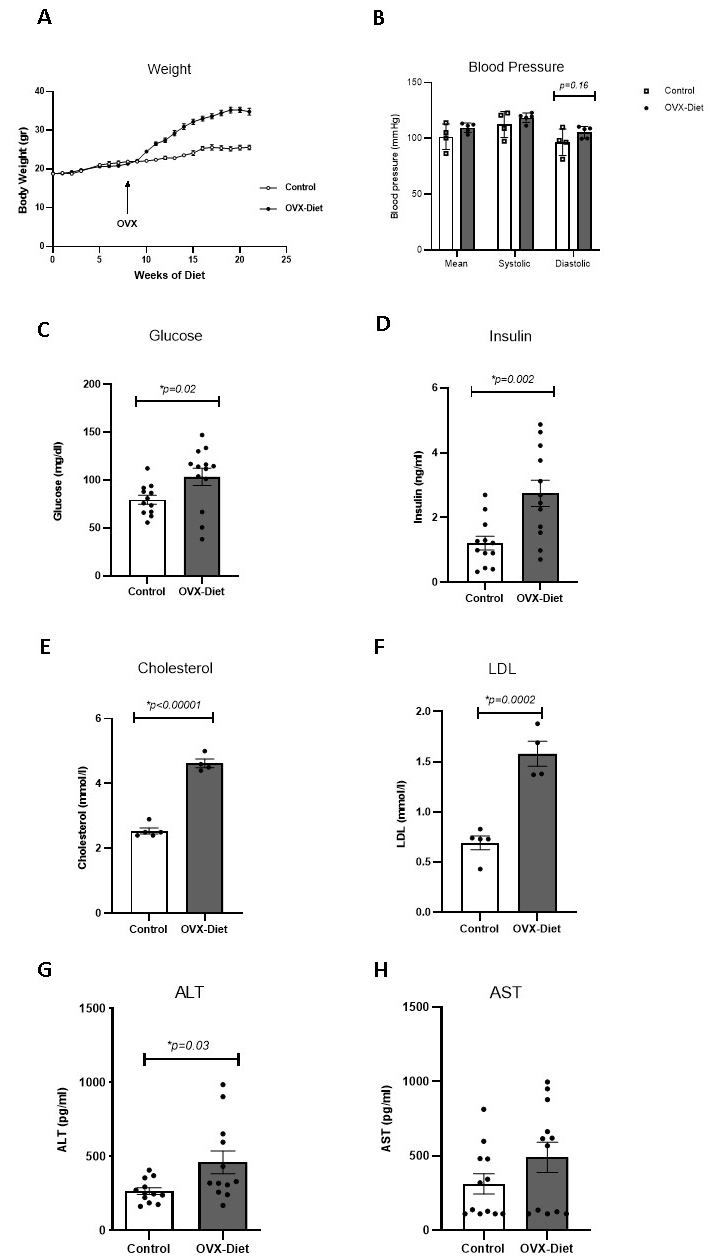
Increased weight, blood pressure, serum glucose and insulin, lipids, and liver enzymes in OVX-mice fed a high fat-sugar-salt diet. (A) Higher weight in the OVX-Diet mice than control mice (repeated measures one-way ANOVA for group effect, time effect, and their interaction, p<0.001 for each) (N=12 mice/group). (B) Tendency of increased blood pressure in OVX-Diet mice relative to control mice: systolic, diastolic and mean blood pressure (p=0.29, p=0.16, p=0.18, respectively; t-test; N=4-5/group) (C) Increased glucose level in the serum compared to the control mice (Mann-Whitney) (N=12-13 mice/group). (D) Increased insulin level in the serum (t test) (N=12 mice/group). (E-F) Increased total cholesterol and LDL (serum of 12-13 mice/group were pooled into N=4-5 pools/group in the OVX-Diet mice relative to the control mice (t test). Cholesterol range: 4.5-5 and 2.4-2.9 nmol/l; LDL range: 1.37-1.88 nmol/l and 0.43-0.83 nmol/l, in OVX-Diet vs control, respectively. (G-H) Increased level of the liver enzyme ALT in the in the OVX-Diet mice relative to the control mice and AST (Mann-Whitney) (N=12 mice/group).

### Statistics

The data are presented as mean ± SEM. Data were analyzed using the unpaired two tailed t-test comparing the OVX-Diet group to the control group. For comparison of the body weight along time between the groups - we used the repeated measures one-way ANOVA model for testing group effect, time effect and the interaction between them (IBM SPSS Statiatics version 28). For analysis of the Y maze: in order to appreciate the fact that errors associated with percentage values (such as percentage of success of direct alterations or, conversely, of direct revisits) are inverse to the denominator (in this case the total number of entries each mouse had made), analysis has been carried out using ANOVA with the total number of entries that each mouse had performed during the test used as analytic weight. Analysis was carried out in Stata 16.0 (StataCorp LLC, USA). Results were visualized with Prism 9 (GraphPad Software, USA). Kolmogorov-Smirnov test was performed as a normality test. Whenever normality of data was confirmed student’s t-test was used. The non-parametric alternative Mann-Whitney was applied when data violated assumptions of normality. In Y Maze - normality was assessed with D'Agostino&Pearson test.

We used the term “trend” aiming to describe results with weak evidence, similar to the definition in VSNI (data science software and experimental design software for biosciences, https://vsni.co.uk/blogs/what-is-a-p-value; accessed on 14 February 2023). Statistical significance was accepted at p < 0.05 (*) and trends at p < 0.1 (^) [39].

**Figure 3.**
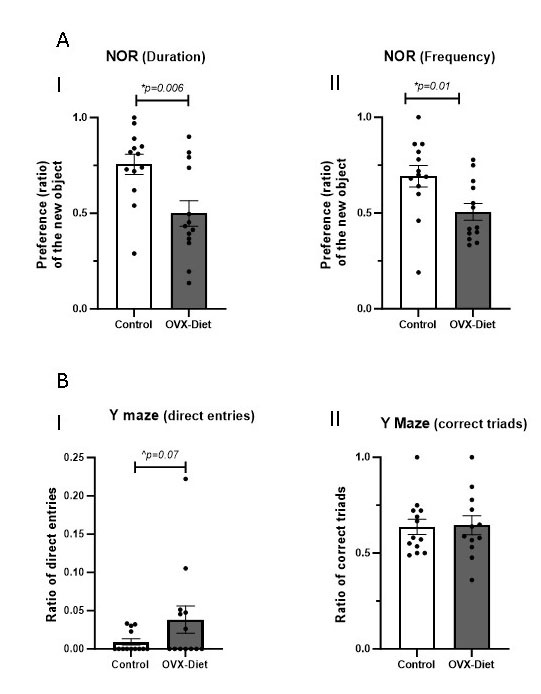
Indication for cognitive impairment in OVX-mice fed a high fat-sugar-salt diet. (A) Novel object recognition test. While the control mice showed a stronger preference for the novel object, the OVX-Diet mice did not show such preference. This was evident in the ratio of both (I) the duration near the novel object (t-test) and (II) frequency (Mann Whitney). (B) Y-maze test. (I) The OVX-Diet mice showed a trend of higher direct revisits index than the control mice (t-test). [Since the control group showed normal distribution, while the OVX-Diet group did not, Mann Whitney test was also performed, revealing also such a tendency, p=0.20). (II) No difference was noted in the correct triads ratio (N=12-13/group). (ANOVA weighted by total number of arm entries, *statistical significance; ^trend).

## RESULTS

### Increased body weight, serum glucose and insulin, lipids, and liver enzymes in OVX-mice fed a high fat-sugar-salt diet

Body weight increased in the OVX-Diet group relative to the control mice (p<0.0001). Weight over time increased in both groups, yet in the OVX-Diet, from week ten onwards, the increase was much faster (p<0.0001), as expressed by a significant interaction between time and group (p<0.0001). The OVX-Diet mice reached a weight of 35.25±0.65 gr, while the control mice reached 25.57±0.6 gr (Fig. 2A).

Blood pressure, measured 5.5 months after starting the diet, showed a tendency to increase in the OVX-Diet mice compared to the control mice: diastolic pressure was 105.58±2.34 mmHg in the OVX-Diet, and 96.62±5.91 mmHg in the controls (p=0.16); systolic pressure was 118.70±1.91 and 112.56±5.84 mmHg, respectively (0.29); mean pressure was 109.58±1.86 and 101.64 ±5.72 mmHg, respectively (p=0.18) (Fig. 2B).

Six months after starting the diet, OVX-Diet mice showed a significant increase in serum glucose compared to the control mice: 103.4±4.5 mg/dl and 79.5 ±9.3 mg/dl, respectively (p=0.02) (Fig. 2C). An increase in serum insulin was also detected in the OVX-Diet mice relative to the control mice: 2.74± 0.4 ng/ml and 1.2 ±0.2 ng/ml, respectively (p=0.002) (Fig. 2D). This points to a glucose/insulin homeostasis dysregulation due to exposure to the diet and OVX.

An increase in total serum cholesterol, about five months after starting the diet, was detected in the OVX-Diet mice relative to the control mice: 4.625 ±0.13 nmol/l and 2.54 ±0.09 nmol/l respectively (p<0.0001) (Fig. 2E); LDL also increased, compared to the control mice: 1.58±0.12 nmol/l and 0.692 ±0.06 nmol/l, respectively (p=0.0002) (Fig. 2F). These results suggest lipid homeostasis dysregulation in response to the diet and OVX.

Analysis of serum liver enzymes, about six months after starting the diet, showed a significant increase in the levels of the ALT in the OVX-Diet mice relative to the control mice: 460.2±79 pg/ml and 265.7±22 pg/ml, respectively (p=0.033) (Fig. 2G). There was also a tendency to increase in the AST level: 490 ±106 pg/ml and 312± 68 pg/ml, respectively (p=0.24) (Fig. 2H). These results indicate liver function dysregulation in response to the diet and OVX.

### Indication for cognitive impairment in OVX-mice fed a high fat-sugar-salt diet

OVX-Diet mice showed significantly lower performance in the novel object recognition test compared to the control mice: while control mice showed a stronger preference for the novel object, the OVX-Diet mice did not show any preference for it [duration ratio: 0.75±0.05 and 0.49±0.06, respectively, (p=0.006); frequency: 0.69±0.05 and 0.50±0.04, in OVX-Diet mice and control mice, respectively, (0.01)] (Fig. 3A). A trend for impaired cognitive performance was also detected in the Y-maze test when analyzing the direct revisits. More direct revisits (higher index) in the OVX-Diet compared to the control mice (0.038±0.018 and 0.009±0.004, respectively) (p=0.07) was recorded (Fig. 3BI). No difference was noted in the correct triad analysis (Fig. 3BII).

**Figure 4.**
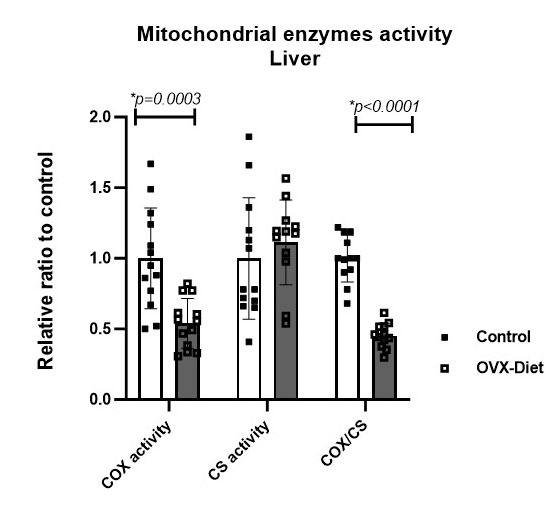
Reduced mitochondrial respiratory chain activity in the liver of OVX mice fed a high fat-sugar-salt diet. Reduced COX activity and COX/CS ratio in the OVX-Diet mice compared to the control mice, with some non-significant increase in CS activity (t-test) (N=13 mice/group).

Testing whether the OVX-Diet mice show motor deficits, we analyzed the distance moved by the mice in te open field, and also in the arena of the Y maze and the NOR test. No significant difference in distance travelled was noticed between the OVX-Diet mice and the control mice: open field (1985±130.1 vs 1765± 83.36, respectively, t-test, p=0.168), Y-maze (1542 ± 121.8 vs 1335 ±- 155/8, respectively, t-test, p=0.301), and NOR (570.1 ± 51.78 vs 478 ± 37.26, respectively, t-test, p=0.159).

Testing for anxiety in the open field test did not indicate increased anxiety in the OVX+diet compared with control mice (time spent in the center of the arena: 25.37 ± 5.52 vs 18.17 ± 4.47, respectively, Mann-Whitney, p=0.31). These results indicate that not only OVX+diet mice did not display increased anxiety, but some opposite effect was noted (which might indicate reduced awareness of the OVX-Diet of the surrounding situation), yet without statistical significance.

No difference in wellbeing of the mice was noticed between the OVX-Diet and control groups.

**Figure 5.**
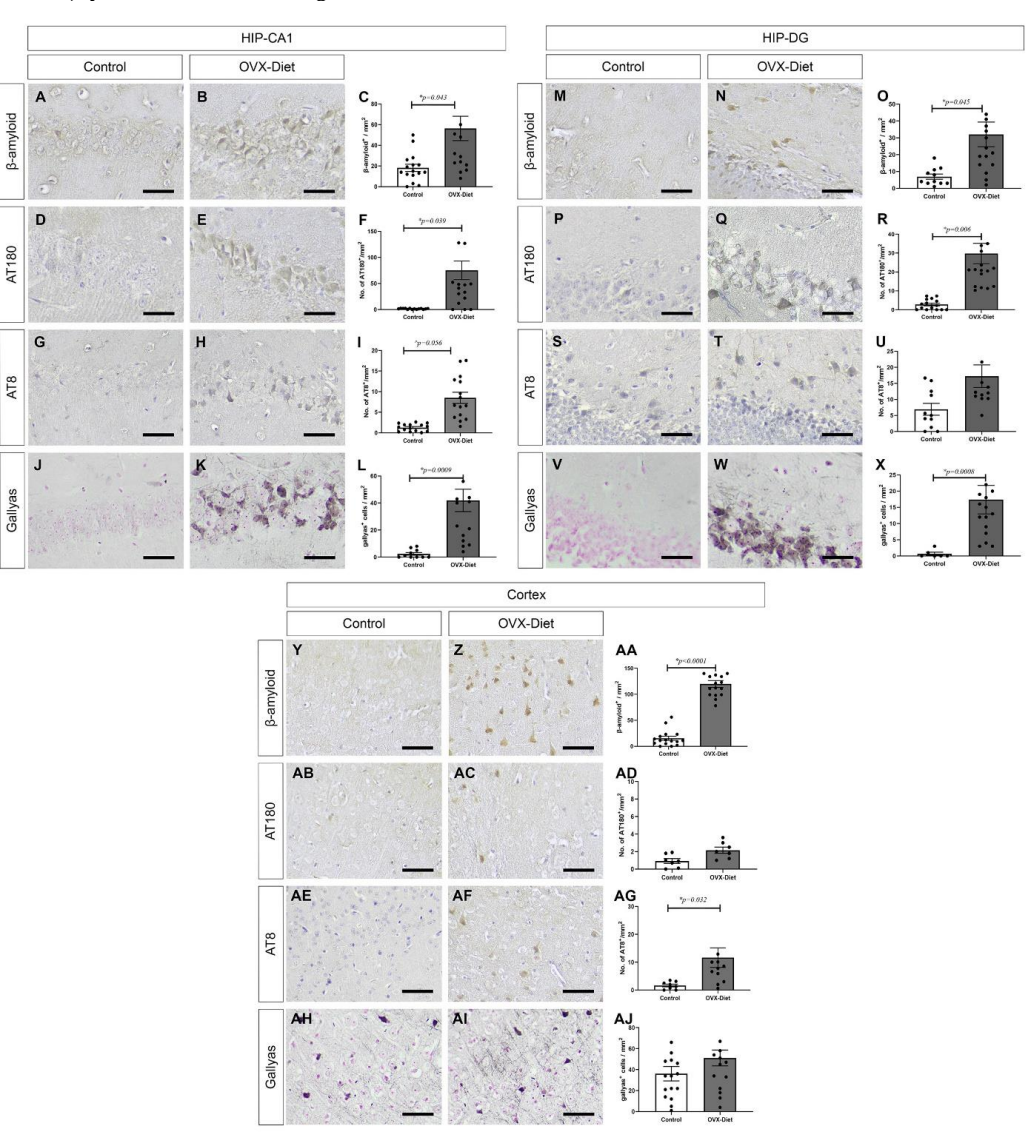
β-Amyloid and tangle pathology in the brain of the OVX-mice fed a high fat-sugar-salt diet. Development of intracellular β-Amyloid and tangle pathology in OVX-Diet mice relative to control mice evident (A-L) in the hippocampus CA1, and (M-X) in the hippocampus DG: (A-C, M-O) β-amyloid, (D-F, P-R) phosphorylated tau-AT180, (G-I, S-U) phosphorylated tau-AT8, tangles -Gallyas staining, (J-L, V-X) (N=7 mice/group), and (Y-AJ) in cortex: (Y-AA) β-Amyloid (N=7 mice/group), (AB-AD) phosphorylated tau-AT180 (N=5 mice/group), (AE-AG) phosphorylated tau-AT8 (N=6 mice/group), tangles -Gallyas staining (AH-AJ) (N=7 mice/group). T-test: * significant, ^ trend. Scale bar = 100um.

**Figure 6.**
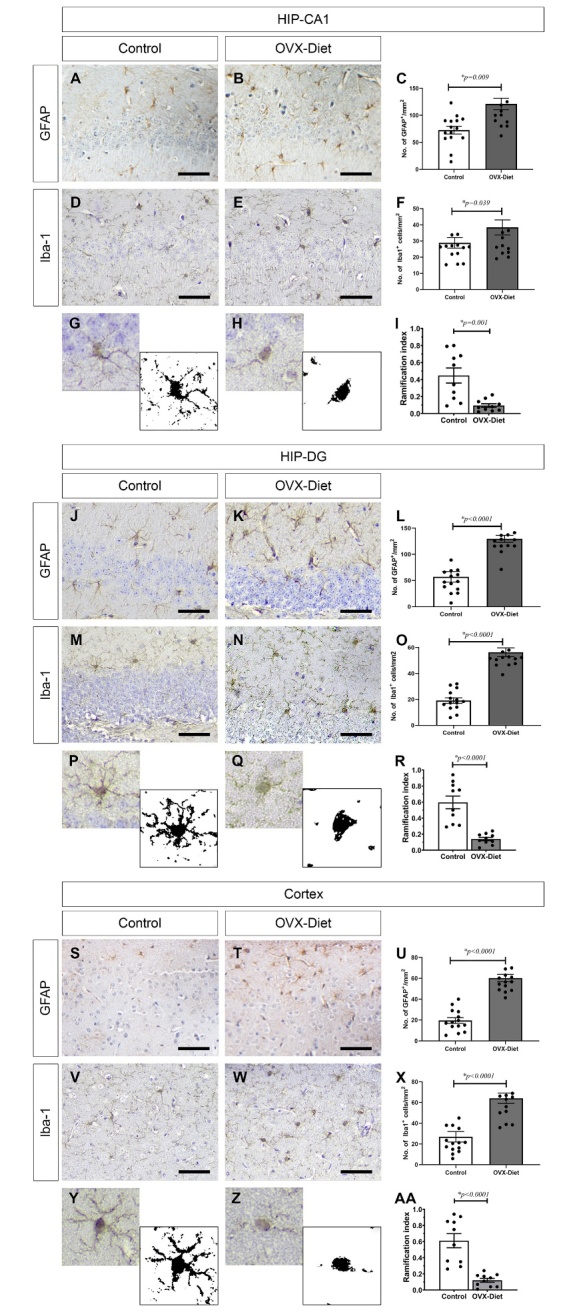
Gliosis in the brain of the OVX-mice fed a high fat-sugar-salt diet. Astrogliosis (GFAP) and microgliosis (Iba-1) in the OVX-Diet mice relative to the control mice evident in the (A-I) in the hippocampus CA1, (J-R) in the hippocampus-DG, and (S-AA) in the cortex: (A-C, J-L, S-U) increased GFAP burden (N=7 mice/group), (D-F, M-O, V-X) increased Iba-1 burden (N=7 mice/group), and (G-I, P-R, Y-AA) activation of microglia (reduced ramification index) (N=5 mice/group). T-test (C, F, L, O, U, X)-, Mann-Whitney test (I, R, AA): * significant., Scale bar = 100um.

### Reduced mitochondrial enzymatic activity in the OVX-mice fed a high fat-sugar-salt diet

We asked if mitochondrial respiratory chain activity is affected in the OVX-Diet mice. The activity of the COX enzyme in the liver was significantly lower in the OVX-Diet mice than in the control mice (about 54%, p=0.0003), and also the COX/CS ratio was lower than the control (about 45%, p<0.0001). No decrease, and even some non-significant increase (11% relative to control), was noted in the activity of CS, a marker of mitochondrial density. This finding suggests that the decrease in the mitochondrial respiratory chain activity, indicated by COX activity, is not related to a reduced number of mitochondria and may even be associated with a compensatory attempt of the liver to increase the mitochondria number by biogenesis (Fig. 4). Since liver insufficiency may induce brain dysfunction [40, 41], and since mitochondria impairment is involved in neurodegeneration-related processes [32, 42-44], we next tested whether the reduced mitochondrial enzyme activity detected in the liver of the OVX-mice was accompanied with reduced activity in the brain. The activity of the mitochondrial enzymes in the cortex of the OVX-Diet mice showed about 10% reduced COX activity and 8% reduced COX/CS ratio relative to the control mice (not statistically significant, p=0.59 in Mann Whiteny and p=0.57 in t test, respectively; N=13/group).

### Amyloid, tangle pathology, gliosis, and vascular impairment in the OVX-mice fed a high fat-sugar-salt diet

We analyzed the brain sections for the presence of AD-related neurodegenerative features. Staining with the anti- β-amyloid Ab detected intracellular amyloid pathology in the hippocampus of the OVX-Diet mice, while control mice hardly showed staining (p=0.043 and p=0.045 in the CA1 and DG, respectively) (Fig. 5A-C, M-O). No staining was detected with Thioflavin-S (data not shown), indicating that the amyloid accumulated is non-fibrillar and did not reach the mature status of amyloid pathology, as plaques. This points that the OVX-Diet mice develop early stage of intracellular amyloid pathology, which may share some similarity with the amyloid pathology reported in very young 5XFAD tg mice [11].

We also detected tangle pathology in the hippocampus of the OVX-Diet mice relative to the control mice, as presented by staining with the AT8 and AT180 Abs for phosphorylated tau and with the Gallyas staining for neurofibrillary tangles (AT8: p=0.056 in CA1; AT180: p=0.039 and p=0.006 in CA1 and DG, respectively; Gallyas: p=0.0009 and p=0.0008, in CA1 and DG, respectively. Staining was barely detected in control mice) (Fig. 5D-L, P-X).

To investigate the occurrence of neuroinflammation features in the brains of the OVX-Diet mice, we stained the hippocampus with Iba-1 and GFAP to detect gliosis. A significantly increased astrocytic burden (GFAP stained cells) was seen in the OVX-Diet mice compared to the control mice (p=0.0009 and p<0.0001 for CA1 and DG, respectively). Additionally, an increased microglial burden (Iba-1 stained cells) was detected in the OVX-Diet mice relative to the control mice (p=0.039 and p<0.0001 for CA1 and DG, respectively). Comparing the RI, a morphometric measure of microglial activation state, revealed a significant increase in microglial activation in the OVX-Diet relative to control mice, presented as decrease in ramification of the microglia (Fig. 6A-I, J-R).

Similar amyloid and tangle pathology and gliosis in the OVX-Diet mice compared to control mice were detected in the cortex. (anti-β-amyloid: p<0.0001, AT8: p=0.032, GFAP: p<0.0001, Iba-1: p<0.0001. The differences in Gallyas and in AT180 did not reach a statistical significance (Fig. 5Y-AJ, Fig. 6S-AA).

**Figure 7.**
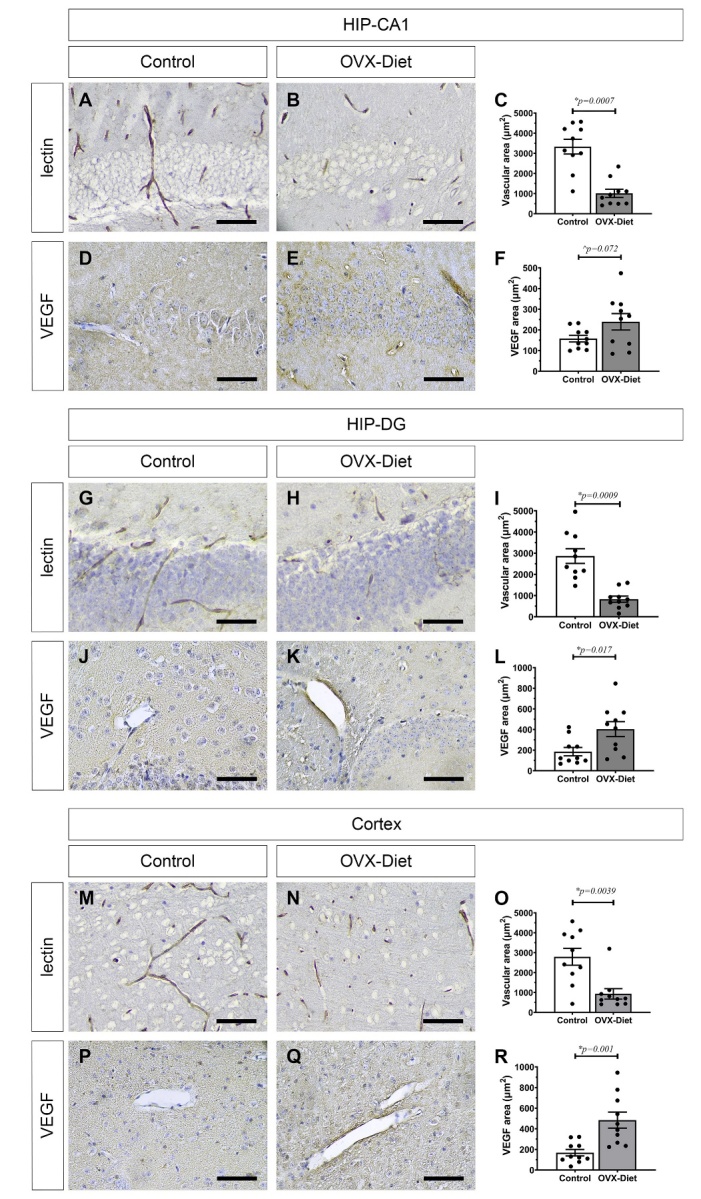
Vascular impairment in the brain of OVX-mice fed a high fat-sugar-salt diet. Vascular impairment in blood vessel density (lectin) and neoangiogenesis (VEGF) in the OVX-Diet mice relative to the control mice evident (A-F) in the hippocampus CA1, (G-L) in the hippocampus-DG, and (M-R) in the cortex: (A-C, G-I, M-O) reduced lectin staining and (D-F, J-L, P-R) increased VEGF staining. (N=5 mice/group). Mann-Whitney test: * significant, ^ trend. Scale bar = 100um.

We next asked if there was evidence of vascular damage in the brains of OVX-Diet mice. Staining with lectin for blood vessels revealed a significant decrease in blood vessel density, expressed as a reduced vascular area in the OVX-Diet mice relative to the control mice. This phenomenon was evident in the hippocampus (p=0.0007 in CA1, and p=0.0009 in DG) (Fig. 7A-C, G-I), and in the cortex (p=0.0039) of OVX-Diet mice (Fig. 7M-O). Similar results were obtained when expressing blood vessel density as the number of vessels per area (data not shown). We also stained for VEGF signal for neoangiogenesis, and detected alterations in the OVX-Diet mice as compared to the control mice, in a manner that there was an increase in VEGF area in the OVX-Diet mice, evident in the hippocampus (p=0.072 in CA1, and p=0.017 in DG) (Fig. 7D-F, J-L), and in the cortex (p<0.001) (Fig. 7P-R).

## DISCUSSION

While AD in most patients is non-familial, the animal models commonly used for studying disease pathogenesis and therapy development are mainly familial, carrying the rare mutant alleles of APP and PS. These models indeed present AD-characteristics, mainly of the amyloid pathology, yet other abnormalities are lacking, particularly neurodegeneration and the tau tangles pathology [45, 46], which correlate best with dementia [47, 48]. Although the efficacy of various drugs for AD has been shown in those animals, they were not effective in the clinic [12, 13].

We aimed to develop a model reminiscent of the etiologies related to the common LOAD sporadic disease that will recapitulate AD/dementia features. Such a model could provide a valid and useful tool for studying disease pathogenesis and developing therapeutic approaches not limited to rare familial AD. Our strategy of exposing naïve mice to the major non-familial AD risk factors by subjecting menopausal females (mimicking aging by OVX) to AD-acquired risk factors induced by a high far-sugar-salt diet revealed brain pathology of amyloid burden, neurofibrillary tangles, and increased glial burden with activated microglia, along with cognitive impairment. An increase in body weight, glucose, and insulin levels, as well as lipids in the serum, with a tendency of increase in blood pressure, was evident.

The lipid homeostasis dysregulation, as well as the increase in body weight, demonstrated here in the OVX-Diet group seems to be attributed to both, the high fat diet that the animals were exposed to, as mice fed a high fat diet have increased body weight and changes in lipids in the serum [49, 50], as well as to the OVX, reported to affect the serum lipids under high fat diet but also to some extent also in normal diet [51]. The lipid dysregulation in OVX mice and in high fat diet fed mice are suggested to be associated with changes in microbiome [51-53].

Our non-familial AD mouse model, which represents a broad spectrum of characteristics of the common sporadic form of AD, also shows vascular impairment. Interestingly, while a decreased blood vessel density (by lectin staining) was detected in the OVX-Diet mice relative to the control, there was an increased neoangiogenesis (by VEGF staining). The increased neoangiogenesis can point to an attempt to overcome vasculopathy taking place in the OVX-Diet mice.This increase in VEGF function is in accord with the reports of pathologically signal of VEGF in AD [54-56], which may be mediated by increasing the BBB permeability, as was reported to take place in depression - with the VEGF/VEGF receptor 2 (VEGFR2) playing a crucial role [57]. Moreover, blocking VEGF function alleviated early-stage cerebrovascular dysfunction and improved cognitive function in a mouse model of Alzheimer's disease [58].

As the AD pathology commonly coexists with that of the vascular dementia, with many of the patients actually being affected by mixed dementia (AD and vascular) [14, 15], the presence of both pathologies - amyloid/tau and vascular impairment- makes our model also representative of AD mixed with vascular dementia.

The OVX-mice fed with a high fat-sugar-salt diet showed reduced mitochondrial respiratory chain activity in the liver, with some similar yet not significant results in the brain. Exposure to components of the Western diet has been reported to cause abnormalities of the liver in the form of increased organ weight [59] and risk of nonalcoholic fatty liver disease [60]. We showed, under high fat-sugar-salt diet in OVX-mice, dysfunction of the liver presented as mitochondrial damage and elevated levels of serum ALT and AST, markers of a liver damage. We can assume that liver dysfunction can affect brain function, as it is the case in hepatic encephalopathy, a brain dysfunction caused by liver insufficiency, and in the nonalcoholic fatty liver disease associated with cognitive changes and brain volume reduction [40]. The communication of the liver and the brain can occur via different routes, such as vagal afferent projections and monocyte migration, with an endocrine, metabolic liver-brain axis [41, 61]. We recently suggested such a crosstalk in intravenous (IV) mitochondrial transfer therapy in AD-transgenic mice. IV transfer of exogenous mitochondria induced a liver response followed by regulated secretion of neuroprotective metabolites into the circulation, reaching the brain via a liver-blood-brain axis [19]. The significantly reduced mitochondrial enzymatic activity detected in the liver of our OVX-Diet mice were accompanied with some non-significant decrease of the mitochondrial activity in the brain. The possibity that some mitochondrial impairment is taking place in the brain, maybe still an early stage, is highly relevant since functional mitochondria are critical for the normal activity of neurons as they are highly dependent on mitochondrial function because of their limited glycolytic capacity [62]. Mitochondria impairment is involved in neurodegeneration-related processes, including apoptosis, energy imbalance, interaction with β-Amyloid and phosphorylated tau-protein, and others [63, 43, 44].

Obesity, diabetes, hyperlipidemia, and hypertension (also part of the metabolic syndrome [65]) are known risk factors for dementia/AD [4, 5]. There is also some evidence in animal studies fed with diets containing single or double-rich components, particularly high fat or sugar, and also high salt [66-70]. A combination of the three rich components, fat-sugar-salt, has been reported particularly for modelling cardiovascular disease, such as vascular stiffness in femoral and mesenteric arteries in C57bl mice fed from four weeks of age for 43 weeks [59], or intramyocardial artery dilation in C57bl mice fed from 3 months for 12 weeks [71]. One study measured the effect of a high fat-sugar-salt diet on the cerebral cortex during aging in 22-month-old male C57bl mice fed for two months. Motor-muscular and sensory dysfunctions were reported with accumulation of amyloid in blood vessels, phosphorylated tau, and microgliosis in the motor and sensory cortexes [72]. In our study, females were fed for about six months from 2 months of age and underwent OVX at 4 months. Surgical menopause depleted the ovarian hormones with their neuroprotective effect [17]. This protocol allowed us to induce an aged status while not requiring the mice to reach old age. Amyloid AD-brain pathology was detected by staining with the anti-β-amyloid Ab showing intracellular pathology. The lack of staining with thioflavin indicates that the amyloid accumulated is non-fibrillar and did not reach the mature status of amyloid pathology, as plaques. This points that the OVX-Diet mice develop early stage of intracellular amyloid pathology, which is similar to the description of intracellular amyloid pathology in the transgenic model of amyloid pathology 5XFAD mice at an early stage [11]. The accumulation of β-Amyloid inside the neurons has been reported to have an important role in the pathogenesis of AD [73]. Also, tau pathology was evident in the OVX-Diet mice, as presented by staining with Abs for phosphorylated tau and by Gallyas silver staining, which stains the AD characteristic neurofibrillary tangle lesions in the brain [34]. We also showed, for the first time, indication for impaired cognition, presented in long term and short-term memory domains. The mice showed increased body weight, glucose levels, insulin and lipids in serum, blood pressure, and liver impairment. These alterations point to pathogenic processes, which are associated with acquired risk for AD/dementia, leading to marked AD/dementia features. Changes in glucose homeostasis or blood pressure were not entirely evident in other mouse studies, possibly due to the use of other high-fat-sugar-salt diets at different ages and periods [71, 72, 59]. Increased plasma glucose levels, even within the normal range (not diabetic), have been associated with decreased processing speed in high-functioning young elderly [74]. This may also be the case with other risk factors, that higher level is possibly associated with a higher risk for dementia, as is the case with cholesterol in cardiovascular diseases, the lower the better for the prevention of disease [75].

Our OVX-Diet mice have the advantage of presenting the common form of AD, in comparison to the familial models represent the rare cases of mutation carriers, and the pharmacological AD models, particularly intracerebroventricular injected β-Amyloid [24], indeed having the advantage of a fast model, yet not representing the chronic slow progressive AD clinical course.

It is not only that the etiology of familial vs sporadic disease differ, but the disease demographics and, of particular interest, the brain pathology- have some differences, including in amyloid and tau burden, and rate of brain atrophy [10]. Importantly, the familial AD mouse models differ not only from the sporadic disease, but they also differ from the familial AD patients, as evident in brain pathology components, particularly the lack of the tangle pathology, a major limitation since tau burden correlate best with dementia, in terms of severity and distribution of the tangles in human brains, better than β-amyloid- plaque burden [47, 48]. Our model has also the advantage that it represents the disease in the context of aging, the strongest risk factor of dementia. As such, it represent specifically the sporadic form in its common late onset, different from another sporadic AD form which is of an earlier age at onset (<65 years, termed sporadic early onset AD, EOAD, which is rare) [10]. Performing OVX (to induce menopause) allowed us to model the disease in the context of the highest risk factor of AD, i.e. aging, yet avoiding the need to use aged animals, waiting for their natural aging. Therefore, a sporadic/environmental model which will not be of the late onset will be less representative of the common sporadic form, which is of late onset. An additional added value of our OVX-Diet model is that it provides us a tool to study the time scale and relationship of development of amyloid vs tau pathology, with challenging which of these pathologies appears first, as it is well established that amyloid pathology precedes the tangles [76], however, the opposite is also taking place - tau aggregation precede diffuse amyloid plaque deposition [77]. In the context of aging and exposure to the diet/risk factors, tau pathology showed a stronger responsiveness than the amyloid pathology, based on the finding that tau pathology reached a mature state (silver staining tangle staining), while the amyloid pathology (at this time point of exposing to the risk factors) - showed the early stage of amyloid pathology, by accumulation of intracellular non fibrillar β-amyloid, yet not developing fibrillar and extracellular plaques. This early stage of amyloid pathology as intracellular accumulation has an important added value for investigating the disease at its early stage, particularly relevant for diagnostic aspects and for early intervention of development. Intracellular amyloid accumulation was also as early stage of amyloid pathology preceding the plaques reported in the young (1.5-month-old, representing age of very young children) 5XFAD tg mice model for familial AD. Having the benefit of using very young mice presenting already this early presentation of amyloid, it may be of value to use mice at stage reminiscent to adults/elderly for potential treatments, as is the case in our OVX adults’ mice. A longer exposure to the diet or an earlier age of OVX may lead to the development of a more mature amyloid pathology. This is actually in accord with the fact that the effect of diseases like diabetes, hypertension, lipid dysregulation- is a slow progressive process and dementia develops after years of being affected by the disease. Therefore, following the model for a longer time may possibly allow us to detect not only more mature amyloid pathology but also more robust cognitive deficits. In support of this, is the disease course of AD, in which the pathology precedes the clinical symptoms [78]. Further research of exposing also naïve animals to each of the AD-related risk factors tested here, OVX and the diet components, separately and in various combinations - would allow to control for the many factors that were affected and would add to the understanding of the contribution of each of them to the phenotype.

To conclude, we show here that naïve mice exposed concomitantly to the major risk factors for AD/dementia, which include aging in females (induced by OVX) and exposure to acquired AD risk factors induced by a high fat-sugar-salt diet, responded by dysregulation of glucose/insulin, lipid, and liver function homeostasis, and by increase in body weight with tendency of increase in blood pressure. The mice developed AD-brain pathology (accumulation of amyloid and tangles), with gliosis, vascular damage, and cognitive impairment, thereby providing a non-familial sporadic/environmental model for AD/dementia with a vascular component. This model may provide us with a model which is more reminiscent to the etiologies related to the LOAD sporadic disease, for studying disease pathogenesis and for the development of therapeutic approaches.
